# NeoNatalie Versus NeoNatalie Live Simulation for Training Undergraduate Students in Neonatal Resuscitation—A Randomized Control Trial

**DOI:** 10.1155/ijpe/3159205

**Published:** 2025-05-22

**Authors:** Anish Sinha, Somashekhar Nimbalkar, Dipti Shah, Purvi Patel, Jaimin Patel, Qury Nagadia, Mayur Shinde, Reshma Pujara, Dipen Patel

**Affiliations:** ^1^Department of Neonatology, Pramukhswami Medical College, Bhaikaka University, Anand, Gujarat, India; ^2^Department of Pediatrics, Pramukhswami Medical College, Bhaikaka University, Anand, Gujarat, India; ^3^Department of Paediatrics, GMERS Medical College, Ahmedabad, India; ^4^Central Research Services, Bhaikaka University, Anand, Gujarat, India

## Abstract

**Background:** The most used manikin for neonatal resuscitation training is NeoNatalie (N), a low-fidelity manikin. A new manikin, NeoNatalie Live (NL), has been developed with more fidelity. We completed a noninferiority RCT to evaluate skill acquisition and to assess retention after 4 months of using these manikins.

**Methodology:** Performance evaluation test (PET), a 14-item checklist, was used to assess students' skills before and after training and after 4 months. The maximum score was 100, and the noninferiority limit was 5. One hundred forty-three medical students were assigned randomly into two groups: N (*n* = 72) and NL (*n* = 71). Half of each group was evaluated on a simulator different from the one they were trained on.

**Results:** Mean (SD) pretest PET scores (before training) for the NL and N groups were comparable across groups (39.5 [18.15] vs. 34.8 [19.10]; *p* = 0.13). The PET score was comparable between NL and N after training (82.46 [10.28] vs. 80.52 [13.07], absolute difference 1.93; 95% CI [−1.956343, 5.830363]; *p* = 0.83 [1-sided]). NL was statistically noninferior to N as the lower bound of 95% CI of absolute difference is greater than the noninferiority margin (−1.95 > −5). A similar finding was observed in retention after 4 months (76.09 [15.80] vs. 73.33 [18.42]; absolute difference 2.75; 95% CI [−2.92457, 8.43271], *p* = 0.83 [1-sided]). The mean gain of PET score within the group (posttest minus pretest) for NL and N was comparable (42.97 [17.11] vs. 45.73 [19.51]; absolute difference 2.76; 95% CI [−8.835228, 3.306668], *p* = 0.81 [1-sided]).

**Conclusion:** There was an improvement in scores in the posttest for both manikins. The NL was noninferior as compared to N.

## 1. Introduction

Timely and efficient neonatal resuscitation plays a crucial role in minimizing mortality resulting from neonatal asphyxia [1]. Effective newborn resuscitation demands the development of cognitive, technical, and behavioral competencies. Prior to 2010, the conventional Neonatal Resuscitation Program (NRP) primarily relied on didactic sessions complemented by skills station training. Nevertheless, employing this instructional approach might lead to inadequate procedural proficiency and resuscitation expertise among numerous trainees during actual rescue scenarios [1].

Since 2010, behavioral skills have been emphasized in neonatal resuscitation. Simulation training has filled this void. “Simulation is a technique, not a technology, to replace or amplify real experiences with guided experiences, often immersive in nature, that evoke or replicate substantial aspects of the real world in a fully interactive fashion” [2]. Designed for educational purposes focusing on the initial segment of the NRP algorithm, bag and mask ventilation (BMV), and corrective ventilation steps, this manikin emphasizes the crucial aspects of training. Learners are unable to perform advanced airway placement and umbilical cord cannulation or receive realistic feedback on chest compressions [3, 4].

The traditional low-fidelity simulation (NeoNatalie) was initially used, but the instructor controlled it. High-fidelity simulators have been developed, but the initial cost and subsequent maintenance are huge barriers to their adoption in resource-poor settings where the need for learning neonatal resuscitation is paramount. In September 2016, a novel manikin named NeoNatalie Live was created as an integral component of the Safer Births project, a collaborative effort involving Laerdal Global Health, Tanzanian, Norwegian, and international research institutions [5]. Designed for educational purposes focusing on the initial segment of the NRP algorithm, BMV, and corrective ventilation steps, this manikin emphasizes the crucial aspects of training. Learners are unable to perform advanced airway placement and umbilical cord cannulation or receive realistic feedback on chest compressions [3]. NeoNatalie Live simulates four different patient cases that reflect the variance in initial heart rate and lung compliance that can happen in real life [5]. The manikin responds realistically to ventilation efforts, with chest rise, increase in heart rate, and baby cry to signal spontaneous breathing after successful resuscitation [5].

Simulation-based learning strategies favor self-efficacy and motivation for learning, affecting clinical skills and knowledge acquisition. These methods can be integrated into educational approaches to enhance active learning [6]. Although meta-analyses of resuscitation simulation studies indicate high effectiveness, there remains additional potential for research on the utilization of simulation as an educational tool [7, 8].

Over the last decade, there has been an increased focus on using simulation methodology for training in newborn resuscitation. Integrating a simulation course into the third-year MBBS curriculum can improve the capacity to handle acute clinical issues in authentic settings. No studies on NeoNatalie Live have been conducted in India. Given the substantial population of medical students and newborns in India, it is imperative to explore the applicability of the NeoNatalie Live manikin in our context. Therefore, we conducted a noninferiority RCT to evaluate the skill acquisition between two manikins and to assess retention after 4 months.

## 2. Materials and Methods

The study received approval from the Institutional Human Research Ethics Committee via letter number IEC/BU/2022/Ex.22/77/2022.

### 2.1. Instrument

A 14-item checklist known as the PET was utilized, employing a 3-point scale (0, 1, and 2). This checklist encompasses four critical skills, all of which are essential and derived from the questions found in the NRP Textbook of the American Academy of Pediatrics (AAP). Trainers were responsible for its implementation. Out of the five trainers, three underwent training on the NeoNatalie Live simulator for 1 day. The trainers were trained on all four scenarios and then further assessed with the help of PET. All four scenarios were already there in the NeoNatalie Live manikin, and they were tailored according to the case difficulty. The trainers possessed neonatal resuscitation certification, with the guidelines from the AAP NRP serving as their primary reference. Each trainer had a minimum of 2 years of experience in conducting NRP training.

### 2.2. Sample Size

Neonatal resuscitation is a mandatory part of the curriculum for final-year medical students. The PET evaluates the postcourse performance. The maximum possible score is 100. The noninferiority limit was set at 5 based on expert clinical judgment. To achieve 90% power and a level of significance of 5%, a sample size of 56 per group (total of 112) is required. Considering a dropout of 5%, a sample size of 60 per group is required. However, as we cannot deny other students' training and considering dropouts, we invited all 143 students to be part of the study.

All participants provided their informed consent.

### 2.3. Methodology

**Figure 1:**
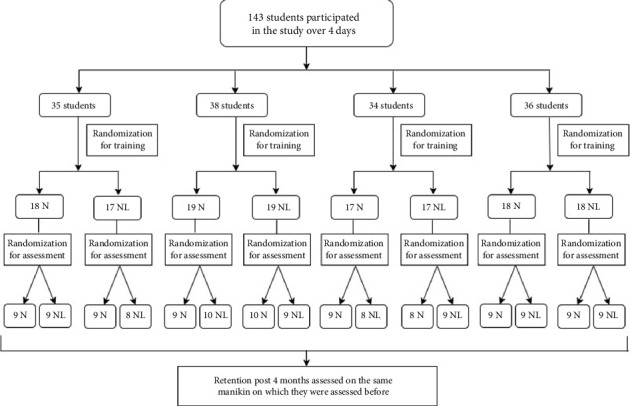
Consort diagram for flow of participants.

One hundred and forty-three final-year medical students were assigned randomly to two groups—NeoNatalie or NeoNatalie Live—and trained over 4 days. They were assessed for skills by instructors on NeoNatalie before training. Then, the students were trained on NeoNatalie or NeoNatalie Live, depending on their randomization. Following their training, half the students in each group were evaluated on a different manikin than the one they were trained on (Figure 1). The remaining half were assessed on the same manikin they were trained on. Randomization was done by computer-generated software every day. The roll numbers of students to be trained every day were fed into the random number generator by the statistician, and the output was used to allocate students to the N or NL groups. For those to be allocated to a different group for assessment, a subsequent randomization was generated so that allocation was random and computer-generated. The statistician was present at the training and ensured the collection of other data related to the study. A postretention test was done after 4 months. The trainers who assessed students were also rotated across the manikins so that the position of the evaluator did not influence evaluation with respect to the manikin. A feedback form was introduced as a Google form comprising 15 questions, which was scanned by the students every day at the time of testing technical and cognitive skills at the end of the day. The questions were designed after discussing the methodology with senior trainers who have been conducting resuscitation training for students for decades. After the questions were developed, they were tested for validation by face validation. The responses were analyzed cumulatively at the end of four training and assessment days.

### 2.4. Statistical Analysis

Descriptive statistics (mean [SD], frequency [%]) were used, before and after PET scores were compared by paired *t*-test. The independent sample *t*-test was used on PET score at the time of assessment, and chi-square and Fisher's exact test were used to test for differences in skill and knowledge acquisition. On paired nominal data (PASS vs. re-evaluate), McNemar's test was used. One-way ANOVA was used to compare PET scores among the groups N-NL, NL-N, NL-NL, and N-N. The participants in the study were randomly assigned, but the teachers evaluating them were not kept unaware of the conditions. The data was presented by the statistician in the form of Groups 0 and 1 instead of using the names NeoNatalie and NeoNatalie Live.

## 3. Results

One hundred forty-three undergraduate medical students were included and randomly allocated to the N (*n* = 72) or NL (*n* = 71) group. Mean (SD) pretest PET scores (before the training) for the NL and N groups were comparable across groups [39.5 (18.15) vs. 34.8 (19.10); *p* = 0.13)]. PET score was comparable between the two groups after training [82.46 (10.28) vs. 80.52 (13.07), (absolute difference 1.93; 95% CI (−1.956343, 5.830363); *p* = 0.83) (1-sided)]. NL was statistically noninferior to N as the lower range of 95% of CI of absolute difference is greater than the noninferiority margin (−1.95 > −5). Paired *t*-test revealed that both groups showed significant improvement in posttest PET scores. The difference in PET scores between the pretest and posttest was calculated. The mean difference between NL and N was not statistically significant [42.97 (17.11) vs. 45.73 (19.51); absolute difference 2.76; 95% CI (−8.835228, 3.306668), *p* = 0.81 (1-sided)] (Table 1).

**Figure 2:**
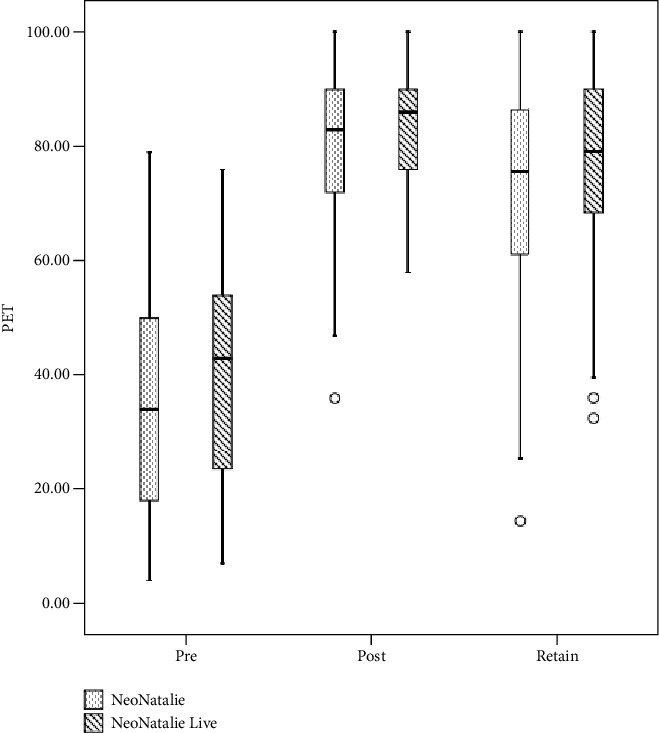
Box plot depicting comparison of PET scores.

A similar finding was observed in retention after 4 months [76.09 (15.80) vs. 73.33 (18.42); absolute difference 2.75; 95% CI (−2.92457, 8.43271), *p* = 0.83 (1-sided)] (Table 1). Box plot depicts the distribution of PET scores between the groups at different time points (Figure 2).

Before the training, no single student from either group passed the PET test. The distribution of students who passed the exam after the activity was comparable between groups NL and N [38 (53.5%) vs. 30 (41.7%); *p* = 0.15)]. When retention was assessed after 4 months, PET scores were sustained in the NL (76.09 (15.8)) and N (73.33 (18.42)) groups, but the difference between the two groups was not significant (*p* = 0.83). Distribution of all the essential steps was comparable between the groups NL and N (Table 2).

The one-way ANOVA test revealed no difference in post-PET scores among the groups N-NL, NL-N, NL-NL, and N-N (the first one is assigned, and the second one is evaluated) [80.7 (13.3) vs. 83.4 (9.2), vs. 81.4 (11.2) vs. 80.3 (12.9); *p* = 0.68]. However, there was a statistically significant difference in PET score after 4 months (retain) between groups as determined by one-way ANOVA (*p* = 0.008) (Table 3).

The Scheffe post hoc test revealed that the PET score in the group assigned and evaluated in NeoNatalie Live (NL-NL) was statistically significantly higher than the PET score in the group assigned and evaluated in NeoNatalie (N-N) (82.15 (11.46) vs. 70.47 (17.87), *p* = 0.037). Similarly, it was higher when assigned to NeoNatalie Live and evaluated in NeoNatalie Live vs. that assigned to NeoNatalie Live and evaluated on NeoNatalie (82.15 (11.46) vs. 70.2 (17.30), *p* = 0.031). Overall evaluation of NeoNatalie Live led to higher scores. However, the four groups had no significant difference regarding PASS and re-evaluate (Table 3).

Feedback was obtained from the students at the time of assessment every day. While the majority of students expressed the helpfulness of the lectures, BMV, routine care, and initial steps were identified as the most crucial topics and the lectures that provided the most assistance. Approximately 61% of students from both groups anticipated remembering over 80% of the content from these lectures in the future (refer to Table 4). Although the number of students who favoured being in the alternate group was higher in NeoNatalie [27 (40.3%)] compared to NeoNatalie Live [17 (28.3%)], this difference was not deemed statistically significant (*p* value 0.15). More students from NeoNatalie Live [58 (96.7%)] wanted additional practice compared to NeoNatalie [64 (95.5%)]. Nevertheless, the observed difference did not achieve statistical significance (*p* value > 0.05). This lack of significance may be attributed to the utilization of the manikin. However, when considering the skills acquired and the confidence in dealing with newborn emergencies in resuscitation, both groups demonstrated comparable outcomes. A total of 75 (59.1%) students regarded neonatal resuscitation as highly significant in their medical careers, particularly in managing and caring for ill or asphyxiated neonates.

## 4. Discussion

This is the first report on using NeoNatalie Live as an educational tool in teaching NRP. Previous studies have objectively examined simulation in neonatal resuscitation [9, 10], yet there has not been a direct comparison between NeoNatalie Live and the NeoNatalie manikin.

Several comparable studies enlisted 15–53 participants [10–13]. In contrast, our study stands out with one of the most substantial participant cohorts, comprising 143 final-year undergraduate medical students.

This study suggests that the extent of improvement in skills acquisition, measured by pass rates and revaluation, does not exhibit significant differences between NeoNatalie Live and NeoNatalie simulation. Additionally, there is no notable distinction in the short-term retention of these skills between the two groups. In a study involving 39 undergraduate students, where participants were divided into four groups (only lecture, lectures+videos, lectures+low fidelity, and lecture+high fidelity), all groups performed equally well in the written test assessing resuscitation knowledge. However, only the lecture-only group performed poorly in the skills testing, while the other three groups showed no significant differences. This led to the conclusion that high-fidelity simulation does not provide substantial benefits to novice students [14]. In a pilot study by Campbell et al. [10], 15 residents were randomized to either a high-fidelity (SimBaby) or traditional plastic manikin (ALS Baby). Similar to our findings, this study demonstrated no significant differences in written evaluation scores or performance task times between the two groups.

Hoadley [12] conducted a study where 53 healthcare providers were randomized to low versus high technical fidelity advanced cardiac life support training. In line with our research, their findings revealed no significant impact on resuscitation knowledge or skills. In a study conducted by Nimbalkar et al., 101 final-year undergraduate students were randomly assigned to low versus high fidelity (Sim NewB). Their results demonstrated no significant difference in learning between the two groups, aligning with the findings in our study [3].

Cavaleiro et al. conducted a study where 45 final-year undergraduate students were randomized into self-study and high-fidelity simulator groups [15]. The comparison involved 30-min supervised self-study versus 30-min neonatal resuscitation sessions utilizing a high-fidelity Gaumard simulator. Their results revealed no distinctions in study scores between the pretest and posttest or between the groups. Notably, the self-study group focused solely on theoretical aspects without simulator sessions. Similarly, in a study by King and Reising [11], where 49 nursing students were randomized to static or high-fidelity simulation, no significant differences were observed between the groups in a written examination. However, the high-fidelity group surpassed the static simulation group in Megacode performance.

We observed no deterioration in skills 4 months posttraining, a deviation from the typical trend. When an integrated learning course spans 2–3 years, the likelihood of skill retention persists until the completion of the internship and even further [16]. In the present study, the absence of skill decline at the 4-month mark could be attributed to the students being in proximity to their final graduation examination. Notably, the 4-month assessment took place less than a month before the graduation assessment, which incorporates a crucial station on neonatal resuscitation for undergraduates. Consequently, their performance remained relatively consistent after the 4-month interval.

Investments and innovations in the simulated learning environment have increased in the last decade. The driving force behind this is the potential for simulation experiences to improve students' learning and engagement. A cost–benefit analysis is important to inform decisions related to the use of simulation equipment [17]. Though the purchase and maintenance cost is more for NeoNatalie Live but the benefits like highly realistic simulation for training and advanced features for complex scenarios, it is worth buying. Furthermore, it improves skill acquisition among healthcare providers. In resource-limited setting, low-fidelity manikin (LFN) might be preferred due to its lower upfront costs and simplicity. But considering the long-term maintenance costs and the frequency of replacements for LFN may impact the overall cost-effectiveness. Still, a detailed cost–benefit analysis is needed, which is beyond the scope of this research.

Although extensive literature exists regarding the application of simulation in resuscitation, there is a scarcity of randomized controlled trials assessing neonatal resuscitation. Furthermore, there are many descriptive studies for NeoNatalie Live but nothing comparing NeoNatalie Live and NeoNatalie in the NRP program. The current study brings newer knowledge to the existing data and can be a benchmark for other institutions to work more on NeoNatalie Live and Simulation. Even more pivotal is determining the impact of simulation training on clinical outcomes. Novel methodologies are imperative for investigating enhancements in clinical outcomes.

## 5. Conclusion

There is a modest improvement in scores trained and evaluated on NeoNatalie Live, which suggests that realistic assessment with higher fidelity may result in better scores. It is a useful tool for teaching NRP to undergraduate students. However, it shows no added advantage over NeoNatalie for retention after 4 months.

## Figures and Tables

**Table 1:** Baseline characteristics, within- and between-group comparisons of NeoNatalie vs. NeoNatalie Live manikin training.

**Variables**	**NeoNatalie Live**	**NeoNatalie**	**p** ** value**	**p** ** value for between-group comparisons using difference scores**
	**n** = 71	**n** = 72		
Gender; male, frequency (%)	31 (43.7%)	44 (61.1%)	—	
Pretest PET scores	39.5 (18.15)	34.8 (19.10)	0.13^d^	0.18^d^
Posttest PET scores	82.46 (10.28)	80.52 (13.07)	0.8365^d^	
*p* value for within-group comparison (before vs. after)^a^	< 0.001	< 0.001		
PET after 4 months (retention) test	76.09 (15.80)	73.33 (18.42)	0.8303^d^	
PET results (pretraining), frequency (%)	0	0	*—*	
Pass				
Re-evaluate				
PET results (posttraining), frequency (%)				
Pass	38 (53.5%)	30 (41.7%)	0.15^b^	
Re-evaluate	33 (46.5%)	42 (58.3%)		
PET results after 4 months (retention), frequency (%)				
Pass	28 (39.4%)	26 (36.1%)	0.68^b^	
Re-evaluate	43 (60.5%)	46 (63.9%)		
*p* value for within-group comparison (after vs. after 4 months)^c^	0.11	0.58		

^a^ *p* values were generated using the paired *t*-test.

^b^ *p* value was generated using the chi-square test.

^c^ *p* value was generated using the exact McNemar test.

^d^ *p* value was generated using the independent sample *t*-test.

**Table 2:** Comparison of PET scores for essential steps.

**Essential steps**	**NeoNatalie Live**	**NeoNatalie**	**p** ** value**
	**n** = 71	**n** = 72	
After training			
Test function of bag and mask	51 (71.83)	48 (66.67)	0.50^b^
Indicates need for PPV	71 (100)	71 (98.61)	> 0.999^a^
Takes corrective step if chest rise is not adequate	70 (98.59)	69 (95.83)	0.620^a^
Ventilates for 30 s and calls for help	67 (94.37)	66 (91.67)	0.52^b^
All essential steps done as mentioned above	50 (70.42))	46 (63.89)	0.40^b^
After 4 months (retention)			
Test function of bag and mask	53 (74.65)	62 (86.11)	0.08^b^
Indicates need for PPV	71 (100)	71 (98.61)	> 0.999^a^
Takes corrective step if chest rise is not adequate	63 (88.73)	58 (80.56)	0.17^b^
Ventilates for 30 s and calls for help	61 (85.92)	59 (81.94)	0.51^b^
All essential steps done as mentioned above	47 (66.20)	47 (65.28)	0.90^b^

^a^ *p* value was generated using Fisher's exact test.

^b^ *p* value was generated using the chi-square test.

**Table 3:** Comparison of PET scores as per allotment and assessment groups.

**PET**	**Allotted assessment**	**NeoNatalie–NeoNatalie Live**	**NeoNatalie Live–NeoNatalie**	**NeoNatalie Live–NeoNatalie Live**	**NeoNatalie–NeoNatalie**	**p** ** value**
Post	Mean (SD)^a^	80.72 (13.35)	83.44 (9.29)	81.45 (11.25)	80.33 (12.97)	0.68
Retain	Mean (SD)^a^	76.2 (18.76)	70.2 (17.30)	82.15 (11.46)	70.47 (17.87)	0.008
PET results (posttraining)	Frequency (%) pass	17 (47.22)	21 (58.33)	17 (48.57)	13 (36.11)	0.31^b^
	Re-evaluate	19 (52.78)	15 (41.67)	18 (51.43)	23 (63.89)	
PET results (after 4 months [retention])	Frequency (%) pass	16 (44.44)	10 (27.78)	18 (51.43)	10 (27.78)	0.08^b^
	Re-evaluate	20 (55.56)	26 (72.22)	17 (48.57)	26 (72.22)	

^a^One-way ANOVA test.

^b^ *p* value was generated using the chi-square test.

**Table 4:** Feedback regarding perception of the course.

**Variable/question**	**Categories**	**Total students' frequency (%)**	**NeoNatalie frequency (%)**	**NeoNatalie Live frequency (%)**	**p** ** value**
		**N** = 127	**N** = 67	**N** = 60	
Would you have preferred to be in the other group?	Yes	44 (34.6%)	27 (40.3%)	17 (28.3%)	0.15

Were the skill stations helpful?	Yes	127 (100%)	67	60	NA

Most helpful skill station?	Bag and mask resuscitation	120 (94.5%)	65 (97%)	55 (91.7%)	0.17
	Routine care	51 (40.1%)	27 (40.3%)	24 (40%)	0.97
	Initial steps	53 (41.7%)	29 (43.28%)	24 (40%)	0.70

Did you get enough time to practice on a manikin?	Yes	122 (96.1%)	64 (95.5%)	58 (96.7%)	0.5

Were the instructors helpful?	Yes	127 (100%)	67	60	NA

How much of the course do you think can you remember?	40%–60%	9 (7.1%)	4 (5.9%)	5 (8.3%)	0.8
	60%–80%	40 (31.5%)	20 (29.8%)	20 (33.3%)	
	> 80%	78 (61.4%)	43 (64.2%)	35 (58.3%)	

Do you think if you are in charge of a newborn with difficulty of breathing at birth you will be independently able to take care of the baby with a person less skilled than you?	Yes	110 (86.6%)	56 (83.6%)	54 (90%)	0.42
	No	9 (7.1%)	5 (7.5%)	4 (6.7%)	
	Maybe	8 (6.3%)	6 (8.9%)	2 (3.3%)	

Would you be interested in doing this course again?	Yes	92 (72.4%)	52 (77.6%)	40 (66.7%)	0.17

Would you recommend this course to your juniors?	Yes	127 (100%)	67	60	NA

In terms of learning neonatal resuscitation, how much useful would it be for helping babies?	< 50%	7 (5.5%)	3 (1.5%)	4 (3.3%)	0.63
	50%–70%	15 (11.8%)	7 (10.4%)	8 (13.3%)	
	70%–85%	30 (23.6%)	15 (22.3%)	15 (25%)	
	> 85%	75 (59.1%)	42 (62.7%)	33 (55%)	

How satisfied were you with the training on a Likert scale of 1–5?	Moderately satisfied	3 (2.36%)	1 (1.5%)	2 (3.3%)	0.42
	Very satisfied	54 (42.52%)	32 (47.8%)	22 (36.7%)	
	Completely satisfied	70 (55.12%)	34 (50.7%)	36 (60%)	

Did you read the basic Nrp before attending the lecture?	Yes	112 (88.2%)	59 (88.1%)	53 (88.3%)	0.9

How many times you revise the basic Nrp algorithm/video?	Never	6	3 (4.5%)	3 (5%)	0.41
	Once	69 (54.3%)	33 (49.2%)	36 (60%)	
	Twice	39 (30.7%)	25 (37.3%)	14 (23.3%)	
	Thrice	13 (10.2%)	6 (8.9%)	7 (11.7%)	
	Completely satisfied	70 (55.12%)	34 (50.7%)	36 (60%)	

## Data Availability

The data that support the findings of this study are available from the corresponding author upon reasonable request.

## Nomenclature

NRP: Neonatal Resuscitation Program
BMV: bag and mask ventilation
LMIC: low- and middle-income country patients

## References

[1] Huang J., Tang Y., Tang J. (2019). Educational Efficacy of High-Fidelity Simulation in Neonatal Resuscitation Training: A Systematic Review and Meta-Analysis. *BMC Medical Education*.

[2] Gaba D. M. (2004). The Future Vision of Simulation in Health Care. *BMJ Quality & Safety*.

[3] Nimbalkar A., Patel D., Kungwani A., Phatak A., Vasa R., Nimbalkar S. (2015). Randomized Control Trial of High Fidelity vs Low Fidelity Simulation for Training Undergraduate Students in Neonatal Resuscitation. *BMC Research Notes*.

[4] Grenvik A., Schaefer J. (2004). From Resusci-Anne to Sim-Man: The Evolution of Simulators in Medicine. *Critical Care Medicine*.

[5] https://shop.laerdalglobalhealth.com/product/neonatalie-live/.

[6] Oh P. J., Jeon K. D., Koh M. S. (2015). The Effects of Simulation-Based Learning Using Standardized Patients in Nursing Students: A Meta-Analysis. *Nurse Education Today*.

[7] Mills D. M., Williams D. C., Dobson J. V. (2013). Simulation Training as a Mechanism for Procedural and Resuscitation Education for Pediatric Residents: A Systematic Review. *Hospital Pediatrics*.

[8] Mundell W. C., Kennedy C. C., Szostek J. H., Cook D. A. (2013). Simulation Technology for Resuscitation Training: A Systematic Review and Meta-Analysis. *Resuscitation*.

[9] Halamek L. P., Kaegi D. M., Gaba D. M. (2000). Time for a New Paradigm in Pediatric Medical Education: Teaching Neonatal Resuscitation in a Simulated Delivery Room Environment. *Pediatrics*.

[10] Campbell D. M., Barozzino T., Farrugia M., Sgro M. (2009). High-Fidelity Simulation in Neonatal Resuscitation. *Paediatrics & Child Health*.

[11] King J. M., Reising D. L. (2011). Teaching Advanced Cardiac Life Support Protocols: The Effectiveness of Static Versus High-Fidelity Simulation. *Nurse Educator*.

[12] Hoadley T. A. (2009). Learning Advanced Cardiac Life Support: A Comparison Study of the Effects of Low- and High-Fidelity Simulation. *Nursing Education Perspectives*.

[13] Donoghue A. J., Durbin D. R., Nadel F. M., Stryjewski G. R., Kost S. I., Nadkarni V. M. (2009). Effect of High-Fidelity Simulation on Pediatric Advanced Life Support Training in Pediatric House Staff: A Randomized Trial. *Pediatric Emergency Care*.

[14] Adams A. J., Wasson E. A., Admire J. R. (2015). A Comparison of Teaching Modalities and Fidelity of Simulation Levels in Teaching Resuscitation Scenarios. *Journal of Surgical Education*.

[15] Cavaleiro A. P., Guimarães H., Calheiros F. L. (2009). Training Neonatal Skills With Simulators?. *Acta Paediatrica*.

[16] Nicol P., Carr S., Cleary G., Celenza A. (2011). Retention into Internship of Resuscitation Skills Learned in a Medical Student Resuscitation Program Incorporating an Immediate Life Support Course. *Resuscitation*.

[17] Lapkin S., Levett-Jones T. (2011). A Cost–Utility Analysis of Medium vs. High-Fidelity Human Patient Simulation Manikins in Nursing Education. *Journal of Clinical Nursing*.

